# Dual Role of Toll-like Receptors in Human and Experimental Asthma Models

**DOI:** 10.3389/fimmu.2018.01027

**Published:** 2018-05-15

**Authors:** Amin Zakeri, Momtchilo Russo

**Affiliations:** ^1^Department of Clinical Medicine, Aarhus University, Aarhus, Denmark; ^2^Department of Infectious Diseases, Aarhus University Hospital, Aarhus, Denmark; ^3^Department of Immunology, Institute of Biomedical Sciences, University of São Paulo, São Paulo, Brazil

**Keywords:** asthma, asthma models, toll-like receptors, bacterial lipopolysaccharide, Th2/Th1/Th17 cells

## Abstract

Asthma is a chronic airway inflammatory disease that is influenced by the interplay between genetic factors and exposure to environmental allergens, microbes, or microbial products where toll-like receptors (TLRs) play a pivotal role. TLRs recognize a wide range of microbial or endogenous molecules as well as airborne environmental allergens and act as adjuvants that influence positively or negatively allergic sensitization. TLRs are qualitatively and differentially expressed on hematopoietic and non-hematopoietic stromal or structural airway cells that when activated by TLRs agonists exert an immune-modulatory role in asthma development. Therefore, understanding mechanisms and pathways by which TLRs orchestrate asthma outcomes may offer new strategies to control the disease. Here, we aim to review and critically discuss the role of TLRs in human asthma and murine models of allergic airway inflammation, highlighting the complexity of TLRs function in development, exacerbation, or control of airway allergic inflammation.

## Toll-Like Receptors (TLRs) at a Glance

Pattern-recognition receptors (PRRs) are a group of innate conserved sensors, which actively contribute to detection of exogenous or endogenous molecules derived from microbes or from host cells. Among PRRs, TLRs play a role in allergic diseases since their relevance in asthma is well documented (1). TLRs ligands derived from pathogens or from host are known as pathogen-associated molecular patterns or damage associated molecular patterns, respectively (1). TLRs are type I transmembrane receptors distinguished by their ligand specificity found on the plasma membrane [TLR1, TLR2, toll-like receptor-4 (TLR4), TLR5, and TLR6] or endosomal compartments of cells (TLR3, TLR7, TLR8, and TLR9) (2). They are expressed by a wide range of cells of the immune system and non-immune cells, such as epithelial cells (ECs) (2). So far, 12 to 10 mouse or human functional TLRs have been identified and each of them is responsible for recognizing a distinct set of molecular patterns.

**Figure 1:**
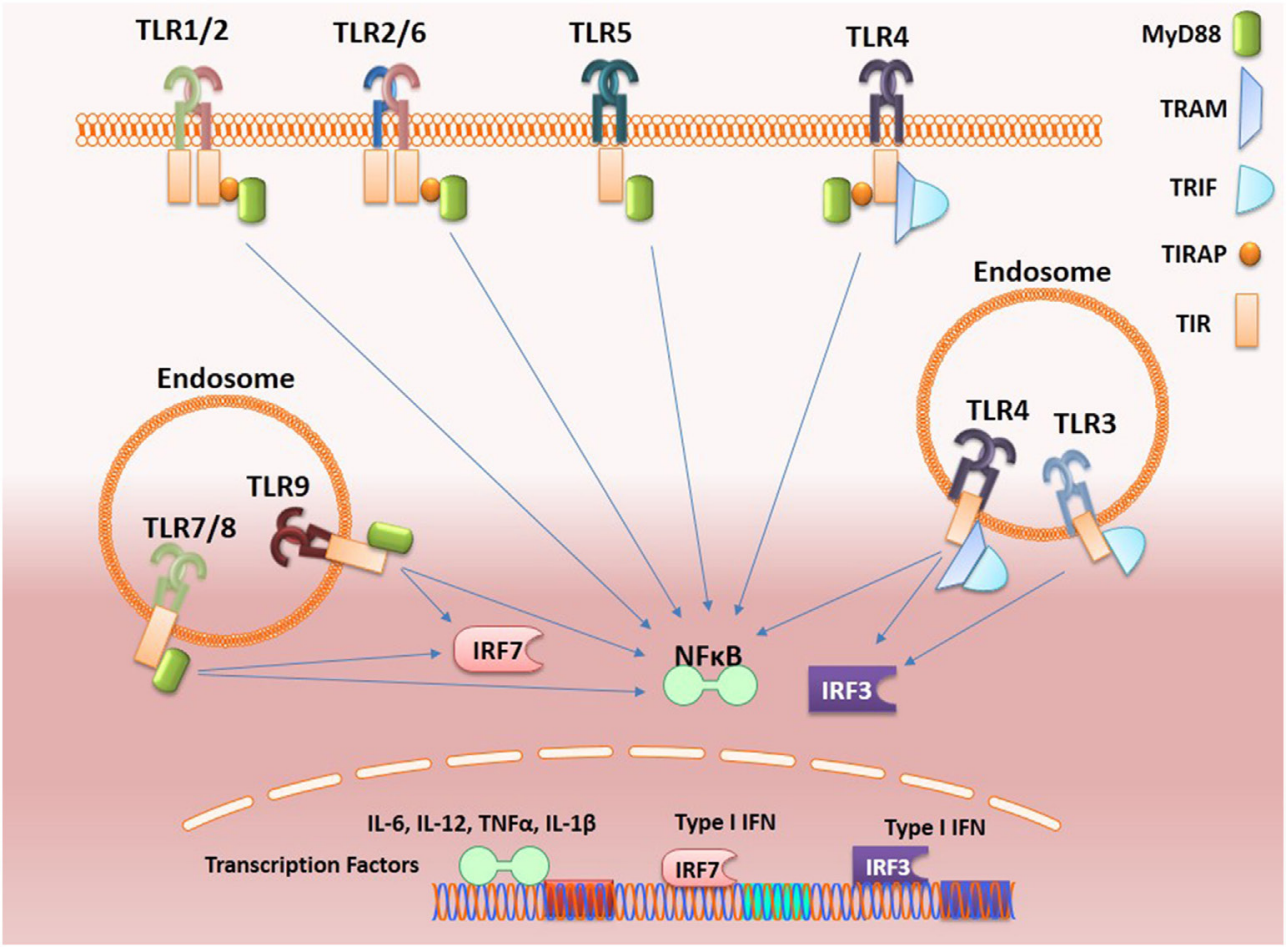
Toll-like receptors (TLRs) signaling. Activation of TLRs can proceed through MyD88-dependent or TRIF-dependent pathways. Most of the TLRs form homodimers upon activation while TLR2 can also form heterodimers with either TLR6 or TLR1. These signals culminating in the activation of transcription factors such as nuclear factor-κB (NF-κB) and interferon-regulatory factors (IRFs), which induce, respectively, the production of inflammatory cytokines and type 1 interferon (IFNs). Activation of endosomal TLRs (TLR7 and TLR9) *via* MyD88 activates NF-κB and also IRF7 leading, respectively, to the production of inflammatory cytokines and type-1 IFNs, while the adaptor protein TRIF is recruited by the endosome-localized receptors TLR3 and toll-like receptor-4 (TLR4). TLR3 can interact directly with TRIF, while the TLR4–TRIF interaction requires the bridging to adaptor molecule TRAM and both activate IRF3 that induce the production of type I IFNs.

As shown in Figure 1, stimulation of TLR complex *via* Toll/interleukin-1 receptor (TIR) domains activates two major signaling pathways, the myeloid differentiation (MyD) 88 and TIR-domain-containing adapter-inducing interferon-β (TRIF). The MyD88-dependent pathway *via* IRAK family kinases mediates induction of inflammatory cytokines *via* nuclear factor-kappa B (NF-κB), mitogen-activated protein kinases, and activator protein 1 while the TRIF-dependent pathway mediates induction of type I interferons (IFNs) *via* interferon-regulatory factors (IRFs). All TLRs except TLR3 that signals through TRIF, recruit MyD88 adaptor molecule while TLR4 activates both the MyD88-dependant and the endosomal TRIF-dependent pathways. Interestingly, stimulation of MyD88 pathway by endosomal TLRs (TLR7, TLR8, and TLR9) results in the production of inflammatory cytokines *via* NF-κB and type I IFNs *via* IRFs.

## Hygiene Hypothesis and Allergic Diseases

The basis of the hygiene hypothesis relies on studies indicating that several microbial products masterfully activate TLRs that, in turn, might exert their suppressive effect against allergic diseases. The postulated inverse relationship between asthma and infections is the basis of the “hygiene hypothesis” (3). Originally, this hypothesis suggested that early-life infections are required for reduced predisposition to develop allergic diseases (4).

Nowadays, the manipulation of TLRs in order to override and control asthma has received considerable attention (5–8). Surprisingly, in contrast with the protective role of TLRs, various allergens have been classified as a third class of TLRs stimuli that participate actively in asthma development (1).

The scenario that emerges from the literature is that TLRs could promote, exacerbate, or ameliorate airway inflammatory response (9, 10).

It is known that Th2 responses are predominant *in utero* and newborn infant that in turn, might predispose to asthma. According to the “hygiene hypothesis,” exposure to microbial agents that drive the immune system to a Th1/Th17 pattern might counterbalance Th2 responses and asthma development (11). In addition, it has been recognized that the diversity and composition of the microbiota of the intestine and lung affect asthma outcome since low diversity in intestinal microbiome has been found to increase asthma development (12, 13).

A growing body of evidence implies that bacteria and parasitic helminths in the digestive tract offer protection against asthma and allergies (14–16). Numerous reports have shown the inhibitory effects of bacterial components on allergic responses (17). Interestingly, the mattress concentration of muramic acid, a constituent of peptidoglycan (PGN) present in Gram-negative and Gram-positive bacteria, was significantly higher in dust from farm children, which showed lower prevalence of asthma and wheezing in comparison to nonfarm school children (16–18). In support of the hygiene hypothesis, it has been reported that the use of antibiotics, which affect commensal bacteria, might be a risk factor for allergic diseases (19). Accordingly, a capsular polysaccharide derived from the commensal bacterium *Bacteroides fragilis* could inhibit murine experimental asthma (20). Bacterial products also exert immunoregulatory effects on asthma development, and it was postulated that the possible mechanism underlying the immunoregulatory effects of bacterial compounds is *via* recruitment of regulatory T (Treg) cells to the airways and the activation of mucosal dendritic cells (DCs) by TLRs-dependent signaling, especially through TLR2/6 and TLR9 (17, 21).

Toll-like receptors agonists are known to act as adjuvant and favor Th1/Th17 responses and they are being used for vaccine development especially against infectious diseases (22).

Interestingly, TLRs agonists have been incorporated in alum-based vaccines to counterbalance the alum Th2 adjuvant activity. For instance, incorporation of monophosphoryl lipid-A, a TRIF-biased TLR4 agonist, to alum has been proposed in alum-based vaccine formulations, such as papillomavirus, genital herpes, and hepatitis B virus (22). Apart from that, the anti-Th2 activity of TLR2 and 4 have received great of interest in the field of experimental asthma (23). In this regard, Bortolatto et al. have shown that incorporation of TLR4 agonists [bacterial lipopolysaccharide (LPS) or synthetic TLR4 agonist (ER-803022)] into alum could suppress the development of allergic Th2 responses without eliciting lung Th1 response (7). In another study Bortolatto et al. showed that addition of LPS to alum-based tetanus toxoid vaccine forestalls toxoid-mediated Th2 responses and IgE production and increases IgG antibody (24). More recently, Mirotti et al. (8) have shown that among different TLR agonists, oligonucleotides (ODN) containing CpG-motifs (CpG-ODN), a TLR9 agonist, was the most effective in dampening all Th2-promoting activities of Alum.

Based on these findings we shall discuss more specifically the involvement of different TLRs in asthma with a special focus on TLR4.

## Plasma Membrane TLR4 and Asthma

Among environmental factors that might positively or negatively influence the development of allergic diseases, endotoxin LPS has gained a particular interest (25). The receptor complex that recognizes LPS includes the LPS-binding protein, CD14, MD2, and TLR4 molecules that are expressed on immune and non-immune cells (26, 27). Typically, signaling *via* TLR4 in innate immune cells results in type 1 cytokine production and consequent development of Th1/Th17 cell immunity (2, 22, 26). In line with this, it is postulated that during early life, TLR4 activation is reduced and the development of Th1 immunity is also reduced favoring the balance toward Th2 responses and susceptibility to develop allergic diseases (28). Therefore, it is surprising that most of the natural allergens such as HDM (house dust mite) not only are contaminated with LPS or PGNs but also have structural homology with TLR4 co-receptors (29, 30). Der p2 as the main component of HDM and responsible for eliciting allergic responses is a lipid binding protein, which structurally has significant similarity with MD2. In fact, both Der p2 and MD2 belong to the similar family of lipid binding proteins. In fact, this structural similarity explains the molecular mechanism of HDM-induced airway allergy. Up to now, this type of structural homology has only been confirmed for Der p2, whereas there are no data concerning the structural relationship between MD2 and other allergens.

## TLR4 Polymorphism and Asthma

To investigate the role of TLR4 in asthma, clinical studies were directed to ascertain whether polymorphism in components involved in TLR4 signaling could be correlated with asthma, atopy, and airway hyperresponsiveness (27). Keeping with this view, some studies supported the notion that allergic inflammation and the regulation of IgE synthesis could be influenced by polymorphism in genes that receptors such CD14, TLR 2, TLR4 among others (27). Also, a link between polymorphism in CD14 gene has been associated with atopy, asthma, and IgE levels (31–33). In contrast, other clinical studies did not support a relationship between polymorphism and the development of asthma (31, 34). Accordingly, it was found that Asp299Gly polymorphism in the extracellular domain of the TLR4 receptor that causes an important reduction of TLR4 function (35) resulted in increased risk to infections (36) or to develop shock (37) but not asthma (38, 39). Similarly, studies considering solely the role of LPS exposure *per se* also reached conflicting results, since some clinical studies demonstrated that exposure to LPS during the childhood could diminish allergic asthma (19, 40) while other studies indicated that LPS could exacerbate allergic asthma (41, 42). For instance in guinea pig asthma model, it has been shown that combination of ovalbumin (OVA) with LPS exacerbates allergic asthma (43).

## Key Factors Involved in TLR4-Mediated Allergic Responses

**Figure 2:**
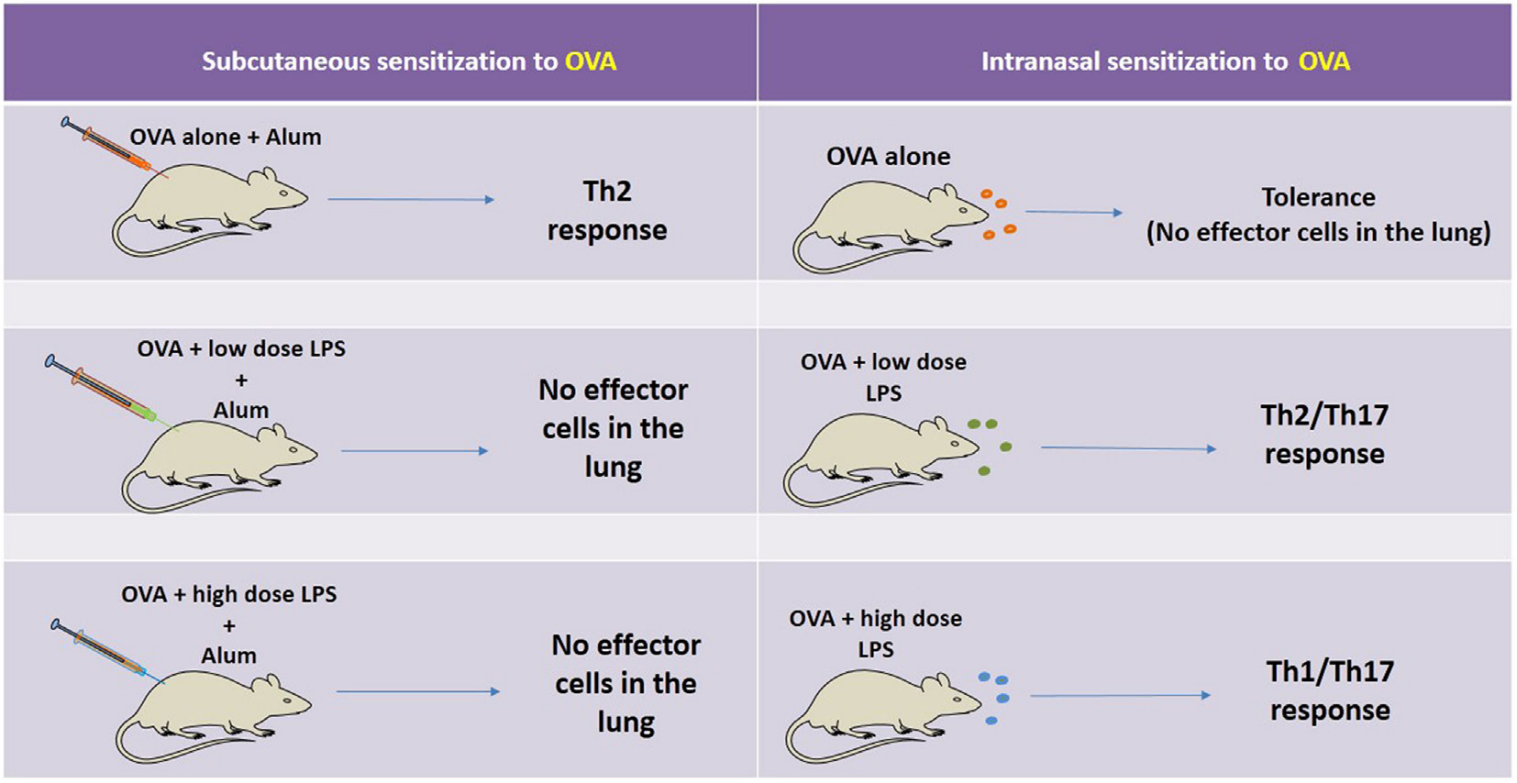
Schematic representation of the factors affecting OVA-model of allergic lung disease. On the left side, it is shown, that when sensitization is performed by the subcutaneous route with OVA adsorbed to Alum and the animals are challenged with intranasal OVA, an eosinophilic airway inflammation mediated by Th2 cells is developed. However, when the same sensitization is induced in the presence of low or high dose of lipopolysaccharide (LPS) and the animals are challenged with OVA, the airway inflammation is not developed. A different scenario is seen with intranasal sensitization to OVA. In this situation, OVA administration results in airway tolerance (no inflammation) while intranasal sensitization with OVA plus low or high dose of LPS results, respectively, in Th2-mediated airway eosinophilia or Th1/Th17-mediated airway neutrophilia. Therefore, part of the controversial reports can be ascribed to the route of sensitization and dose of LPS.

Although the role of TLR4 in asthma is still controversial, it is becoming clear that several factors should be considered in the TLR4-mediated development of allergen-induced Th2 responses, including polymorphism of CD14, the cell type in which TLR4 is engaged, the dose of stimuli, and the timing of exposure (5). The pioneering work of Eisenbarth et al. (44) using a protocol of intranasal administrations of OVA containing low doses of LPS, demonstrated that the presence of TLR4 and MyD88 molecules are necessary for development of allergic response. Histological findings demonstrated that the administration of OVA with low dose LPS increases infiltration of eosinophils and neutrophils, airway mucus secretion, and Th2 cytokines production (44). By contrast, mice exposed to OVA with high dose of LPS showed Th1-associated response, such as airway neutrophilia without mucus secretion and high level of IFN-γ production (43–45). Another study extended these findings and showed that allergic sensitization with low dose of LPS through the airway indeed primes for Th2 responses, but in addition, it also primes for Th17 responses that are essential for promotion of airway neutrophilia and AHR, highlighting the importance of concomitant Th2/Th17 cells in the development of AHR (46). A different scenario was obtained when allergic sensitization was performed with OVA plus LPS administered by intraperitoneal route since it did not result in airway inflammation (46). Similar results were obtained with cutaneous sensitization with OVA plus TLR4 ligand (LPS) or TLR2 ligand (Pam3Cys). In this situation, asthma development and Th2 responses in mice were blocked in an IFN-γ-dependent manner (47). Interestingly, Garcia et al. showed that LPS inhalation before OVA sensitization also blocked Th2 development (48). It was shown that LPS pre-exposition suppressed eosinophil influx and IL-4 production by shaping immune response toward Th1 immunity *via* IL-12 production and classical activation of alveolar macrophages (48). The absorption of OVA to alum adjuvant results in more robust Th2 responses regarding airway eosinophilic inflammation and IgE production (7, 44). Using this OVA-model of allergic lung disease where sensitization to OVA is done by subcutaneous route, it was shown that LPS plus OVA adsorbed to alum suppressed allergic sensitization in a dose-dependent manner, and this suppression was dependent on TLR4 and MyD88 but not TRIF signaling (6). Therefore, part of the controversial findings might be explained whether the route of sensitization is intranasal or subcutaneous/intraperitoneal. The summary of OVA-models discussed above is depicted in Figure 2.

Age is another important factor affecting TLR4-mediated Th2 response by LPS exposure. For instance, LPS exposure of newborn mice inhibits OVA-induced airway inflammation, hyperresponsiveness, and Th2 cytokine expression (49). Also, early exposure to LPS results in development of CD25-positive T cells and IL-10 production in newborn mice, indicating mucosal antigen exposure in the neonatal period may provide tolerance and hyporesponsiveness to environmental allergens (49). In addition, prenatal LPS exposure prevented allergen sensitization in offspring (50). Exposure to bacterial components can modulate immune responses during gestation time and might be an effective way for prevention of allergic diseases. Thus, age and dose of LPS could be key factors in determining protection against asthma.

Mouse models of experimental asthma confirmed that exposure to endotoxin could protect, induce, or exacerbate asthma depending on the experimental model used, route of sensitization, concentration, and period of endotoxin exposure (6, 7, 51–54),.

Fungi-derived allergens especially those with proteolytic activity also have been investigated (55). It was shown that proteinases derived from *Aspergillus* spp., by cleaving airway fibrinogen, contribute to asthma development (55). This study showed that TLR4, but not other TLRs, is essential for induction of airway inflammation by fungal proteinases and that fibrinogen-derived proteins bind to TLR4 on epithelial airway cells and macrophages supporting inflammatory signals and Th2 cell development (55). Therefore, it seems that TLR4 signaling could be pro-allergic when considering airway route.

Contact with small animals such as cats and dogs especially those that are held in house (indoor) can significantly exacerbate asthma through stimulation of TLR4 signaling. Interestingly, the critical function of TLR4 in sensitization with small animal-derived allergens has been established (56). An *in vitro* study has shown that animal danders act in a similar way to lipid binding proteins in stimulation of TLR4 (56).

A key molecule in TLRs signaling is MyD88 and as such MyD88 has gained great of interest as an important adaptor molecule in development of asthma (7, 44). In addition, lack of this critical adaptor protein has been found to prevent the accumulation of inflammatory cells and airway inflammation upon inhalation of HDM (57). Interestingly, in some models, MyD88 molecule appears to be more relevant than TLR4 since MyD88 expression, but not TLR4, was critical for induction of experimental asthma induced by *Alternaria* extract (58). MyD88 is shared by all TLRs except TLR3, as well as by IL-33 receptor, which plays a role in inducing Th2 responses (59). The importance of MyD88 in induction of experimental asthma was further proved when *Alternaria* extract is co-administered with OVA. Lung eosinophilia and production of Th2-related cytokines in response to *Alternaria/*OVA administration was induced *via* IL-33 and MyD88-dependent manner, but independent of TLR4 stimulation (59). Despite the results obtained in TLR4-deficient mice or deficient in downstream molecules indicating that TLR4 signaling is required to induce airway hypersensitivity (44, 57, 60–62), some studies showed that experimental asthma is easily induced in TLR4-deficient or MyD88-deficient mice (7, 8). However, it should be noted that different allergens were employed in these studies suggesting that the nature of the allergens might determine whether induction of allergy is TLR4/MyD88-dependent or independent.

## Interaction Between LPS and TLR4 Expressed by Airway Structural Cells (ASCs)

With the advent of conditional deletion of specific components of TLRs signaling or chimeric mice, the critical role of ASCs in asthma development became clearer. ASCs comprise various cell types including ECs, endothelial cells, and fibroblasts, which play a key role in lung immune responses and airway inflammation (60, 63, 64). Accordingly, stimulation of TLRs in ASCs has been found to drastically affect the sensitization phase. For example, using chimeric mice it has been shown that the stimulation of TLR4 on ASCs by LPS results in airway neutrophilia (60). Also, stimulation of ASCs through a TLR4-dependent manner produces chemokines and G-CSF leading to inflammatory cell recruitment upon exposure to LPS or HDM (63). Another mechanism that enables ASCs-associated TLR4 to support airway neutrophilia is vascular endothelial growth factor (VEGF) which is released upon inhalation of LPS-contaminated allergens (64). Kim et al. demonstrated that intranasal administration of OVA plus LPS induces the release of VEGF, stimulating DCs maturation, upregulation of co-stimulatory markers, and production of IL-12 and IL-6 in regional lymph nodes leading to development of OVA-induced Th1/Th17 lung immunity (64). Interestingly, inhibition of VEGF receptor by pan-VEGF receptor blocker resulted in suppression of Th1/Th17 polarization. This study indicates that intranasal exposure to LPS followed by allergen could forestall the initiation of Th2 response *via* activation of TLR4 on ASCs (63, 64). Furthermore, manipulation of downstream molecules mediating TLR signaling in ASCs corroborates the important role of these cells in airway inflammation. In this regard, Skerrett et al. (65) using transgenic mice with defect in NF-κB activation in airway endothelial cells showed that, upon LPS administration, the airway neutrophilia and production of cytokines, such as TNFα and IL-1β, were impaired when compared to wild-type mice. These data imply that ASCs respond to LPS through activating downstream signaling involving NF-κB, which ultimately leads to release of pro-inflammatory mediators and recruiting neutrophils to the airways (65, 66).

## Effects of TLR4 Signaling on ASCs versus Hematopoietic Cells (HPCs)

Although epidemiological studies demonstrate that high levels of endotoxin exposure may protect against allergen sensitization and inversely correlate with atopic rates (67), it has not been yet formally determined whether TLR4 stimulation in humans promotes or suppresses asthma. Nevertheless, it is known that asthmatic patients express low level of TLR4 in their peripheral blood mononuclear cells PBMCs as compared to healthy individuals. In comparison with healthy subjects, the TLR4 of asthmatic patients produces low level of type 1 cytokines, such as TNF-α and IL-1β, in response to LPS (68). It is likely that factors affecting TLR4–ligand interactions such as agonist concentration and timing of exposure could determine the outcome of pro- or anti-asthmatic effects of TLR4 (69).

As shown in Figure 2, in mouse models where sensitization is performed with alum adjuvant adsorption of TLR4 agonists to alum prevent type-2 sensitization (54) through TLR4- and/or MyD88-dependent but TRIF-independent mechanism (7, 8), indicating that TLR4 signaling during allergic sensitization dampens development of asthma-like responses. In contrast, in OVA-models where allergic sensitization is done by airway route, low dose of LPS promotes Th2 immunity (44).

Now, it is clear that some allergens are able to stimulate TLR4 on ECs causing DC recruitment and maturation (70). Thus, it appears that depending on the dose of LPS, TLR4 signaling on structural lung cells plays a pivotal role in the induction of Th2-associated responses (60). Engagement of TLRs expressed on ASCs by environmental allergens leads to release of mediators that support the development of Th2 responses *via* production of various Th2-associated cytokines, including IL-4, IL-13, and other cytokines, known as alarmins (IL-33, TSLP, and IL-25) (29). Undoubtedly, lung DCs, which have a close interaction with airway ECs are affected by these alarmins and undergo an essential modification required for presentation of allergens to naïve CD4^+^ T cells (71). Airway exposure to LPS or to HDM accelerates the migration of DCs through stimulation of TLR4 on ASCs (60). Also, the release of GM-CSF in response to LPS or HDM by ASCs expressing TLR4 results in DCs activation and priming naïve CD4^+^ T cells (60). It was found that airway inflammation was decreased in mice simultaneously exposed to HDM allergens and TLR4 antagonist when compared to mice exposed to HDM alone (60). Conversely, direct TLR4 signaling on DCs appears to suppress Th2 immunity. Indeed, new insights were gathered recently concerning the distinct role of TLR4 on AECs and HPCs in deviation of immune response toward Th2 or Th1/Th17. Now, it is becoming clear that stimulation of TLR4 on AECs supports Th2 immune responses while stimulation of TLR4 on HPCs suppresses Th2 immunity. In this regard, Hammad et al. (60) by exploiting chimeric mice in which TLR4 expression on ASCs (ASC^−^ HPC^+^) or on HPC (ASC^+^ HPC^−^) was deleted provided solid evidence indicating that the presence of TLR4 on ASCs is essential for activation of pulmonary DCs and initiation of Th2 responses after exposure to HDM. They showed that lack of TLR4 expression on ASCs but with normal expression of TLR4 on HPCs impaired the development of Th2 response (60). Keeping with this view, a similar study indicated that the deviation of immune response toward Th1 or Th2 is dependent on the cell types on which TLR4 is stimulated (61). For example, simultaneous exposure to OVA and high dose of LPS in mice with TLR4 restricted to stromal but not HPCs (ASC^+^ HPC^−^) resulted in strong Th2 response and airway eosinophilia, suggesting stimulation of TLR4 on ASCs is sufficient for priming Th2 response (61). Interestingly, when mice with TLR4 restricted on HPCs (ASC^−^ HPC^+^) were exposed to OVA and high dose of LPS, the resulting immune response was deviated toward Th1 pattern, implying TLR4-mediated signaling on HPCs (immune cells) restrain Th2 response (61). In support of this, McAlees et al. using conditionally mutant TLR4 mice either in airway ECs or HPC showed that exposure of (ASC^+^ HPC^−^) mice to HDM or OVA along with LPS augments eosinophilic airway inflammation (72). The authors confirmed that absence of TLR4 on HPCs (ASC^+^ HPC^−^) results in development of Th2 response and eosinophilic asthma in mice, whereas presence of TLR4 on HPCs but not on ASC (ASC^−^ HPC^+^) supports Th1/Th17 response and neutrophilic asthma (72).

These data reemphasize that TLR4 in the lung is able to mediate distinct arms of immune response upon stimulation by environmental allergens. As discussed above, TLR4 signaling in ASCs instructs DCs in the line of inducing Th2 responses, while this receptor on HPCs is able to trigger signaling pathway that programs DCs to polarize immune response toward Th1/Th17 patterns (25). Keeping with this view, Whitehead et al. investigating the effect of different adjuvants during airway sensitization to OVA found that mice sensitized using TLR ligands or house dust extracts as adjuvants developed mixed eosinophilic and neutrophilic airway inflammation following OVA challenge, whereas mice sensitized using proteases as adjuvants developed predominantly eosinophilic inflammation. The TNF signaling in airway ECs promoted Th2; however, TNF was dispensable for allergic airway disease in a protease-mediated model of asthma (73). Conversely, Schuijs et al. added new information regarding the protective effects of endotoxin in farming environment on the allergic responses. The authors showed that chronic inhalation of endotoxin prior to allergen exposure suppresses EC-derived Th2-promoting mediators (28). Importantly, the Th2-inhibitory effect of endotoxin LPS was not related to the redirection of immune response toward Th1 or Th17 (28). Molecular analysis revealed that A20, which is a ubiquitin-modifying enzyme, plays a pivotal role in the LPS-mediated protective effects against asthma development since mice deficient in A20 in lung ECs exposed to chronic LPS developed HDM-induced Th2-mediated lung inflammation (28). In fact, A20 is able to lower TLRs signaling through deubiquitinating downstream molecules, which eventually blunts the NF-ĸB activation (74). In support to this view comparing healthy patients with asthmatic, it was found that bronchial ECs pretreated with LPS released low level of GM-CSF and IL-1α in response to HDM while in asthmatic patients A20 expression and protein levels were lower than in healthy controls (28). The authors concluded that the main mechanism by which farm dust exerts their protective effects against asthma development is activation of A20 in airway ECs. More recently, the same group using mice with conditional deletion of Tnfaip3 (A20) gene in dendritic cells (DCs) and exposed to HDM developed HDM specific Th17 cell differentiation, through increased expression of Th17-instructing cytokines, IL-1β, IL-6, and IL-23 (75). The authors conclude that A20 levels in DCs critically regulate development of either Th2-mediated eosinophil asthma or Th17-mediated neutrophilic asthma (75).

**Figure 3:**
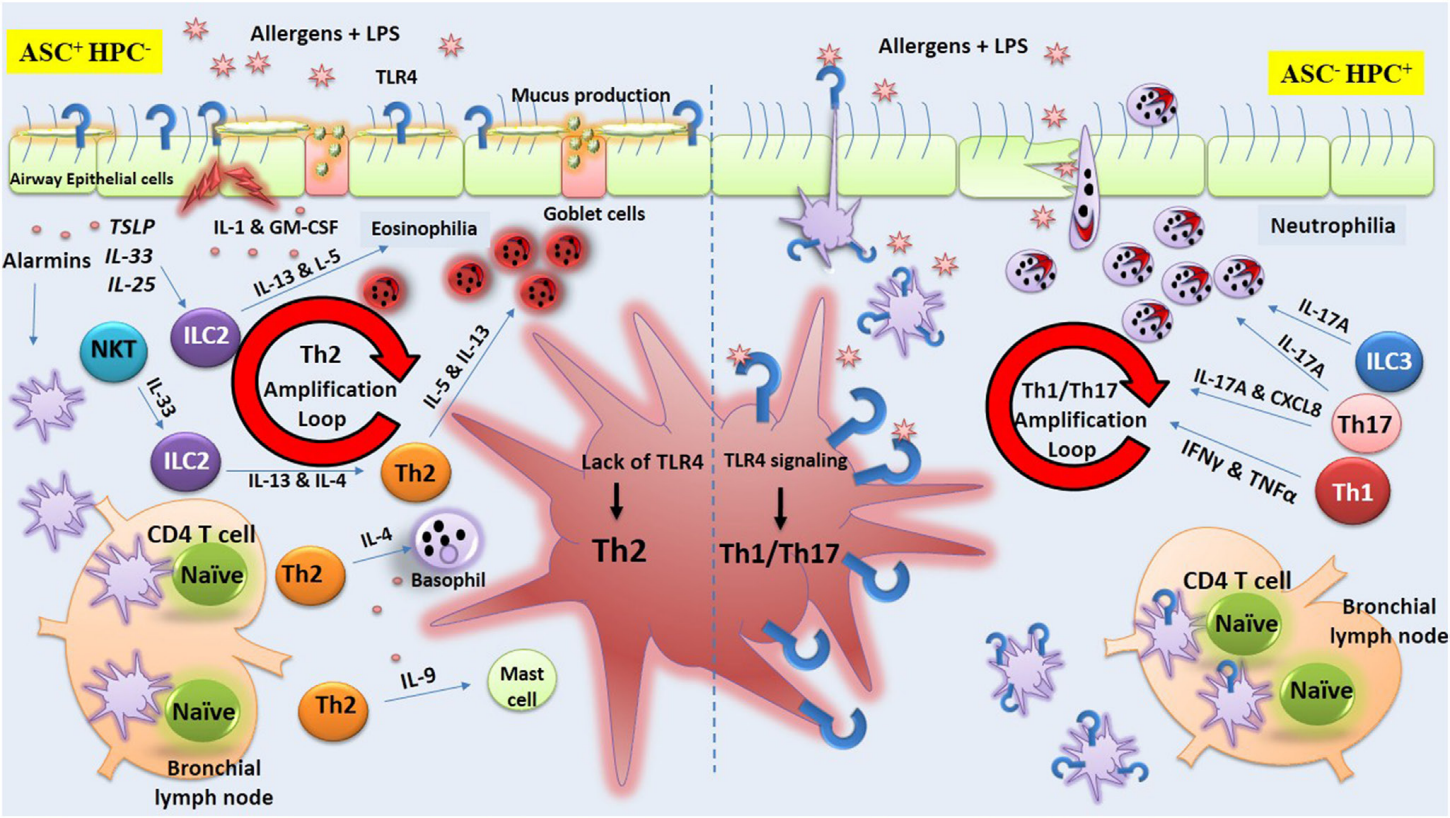
Toll-like receptor-4 (TLR4) signaling on airway structural cells (ASC) versus hematopoietic cells (HPCs). The figure illustrates lipopolysaccharide (LPS)-mediated TLR4 signaling exclusively on ASCs or HPC during allergen exposure that results, respectively, in orientation of immune response toward Th2 or Th1/Th17. The figure is based on studies obtained in chimeric mice showing that stimulation of TLR4 on ASCs supports Th2 immune responses while stimulation of this receptor on HPCs induces Th1/Th17 immunity. On the left side of the figure, it is shown that the lack of expression of TLR4 on HPC but not on airway structural epithelial cell (ECs) (ASC^+^HPC^−^) results in strong Th2 response along with airway eosinophilia. In fact, TLR4 stimulation of ECs (ASC) by LPS or environmental allergens triggers the production of cytokines including alarmins (IL-33, TSLP, and IL-25) that support the development of Th2 responses *via* lung dendritic cells (DCs) or *via* other local lymphoid cells (LLCs). The production of type 2 cytokines orchestrates the activation and recruitment of eosinophils, basophils, and mast cells. In contrast, on the right side of the figure, it is shown that the lack of TLR4 expression on ASCs but not on HPCs (ASC^−^HPC^+^) trigger the production of cytokines that support the development of Th1/Th17 immunity and airway neutrophilia *via* lung DCs or *via* other LLCs.

Altogether, it is clear that various airway cell types are critically involved in the induction or protection against allergic sensitization as well as in immune-deviation toward Th1/Th17 patterns as depicted in Figure 3.

## Other Plasma Membrane TLRs (TLR1, 2, 5, and 6) and Asthma

The role of TLR4 in asthma has been extensively studied while the involvement of other TLRs in asthma has gained less attention. Nevertheless, the studies focusing on the role of other TLRs in asthma also reached divergent results (Table 1). For instance, some studies with TLR1 or TLR2 agonists suggested that their activation results in asthma inhibition (76–78), while other studies indicated that activation of these receptors could promote allergic asthma (79, 80). The pro-allergic effect of TLR2 was emphasized in chronic fungal asthma model induced by i.p. and s.c. injection of soluble *A. fumigatus* antigens dissolved in incomplete Freund’s adjuvant where *Tlr2*^−^*^/^*^−^ C57BL/6 mice showed attenuation of airway hyperresponsiveness, decreased Th2-type cytokines, and suppression of chemokine production when compared to WT mice (81). Likewise, in the OVA-asthma model with s.c. OVA sensitization, addition of TLR2 ligand (Pam3Cys) potentiated allergic sensitization (80, 82) while TLR9 agonist suppressed it (75). The exact mechanism by which TLR2 signaling promotes Th2-associated responses has not yet been fully elucidated. It appears that stimulation of TLR2 expressed on DCs leads to the activation Th2-promoting molecular pathways including the secretion of pro-Th2 cytokines, such as IL-13, IL-1, and GM-CSF (80, 82). In sharp contrast to these findings, TLR2 activation was shown to attenuate allergic airway inflammation in mice exposed to OVA or HDM allergen (8, 23). Keeping with this view, it is believed that augmented TLR2 expression in PBMCs found in children living in farms is the consequence of constant exposure to microbial components that, in turn, protect against allergic responses (83).

**Table 1:** Involvement of TLRs in mouse experimental models of asthma.

TLRs	Allergen	Effect on mouse models of asthma	Main finding
TLR1/2/6	*A. fumigatu*s in incomplete Freund’s adjuvant	Pro-allergic (81)	*Tlr2*^−^*^/^*^−^ C57BL/6 mice showed attenuation of airway hyperresponsiveness, decreased Th2-type cytokines
	
	OVA	Pro-allergic (80, 82)	Addition of TLR2 ligand (Pam3Cys) potentiated allergic sensitization
	
	OVA or Timothy grass pollen	Anti-allergic (86, 87)	TLR2/6 agonist reduced the level of eosinophils, IL-5, IL-4, eotaxin-2 in BALF
	
	Fungal- or HDM antigen	Anti-allergic (88)	TLR6 induced IL-23 production and Th17 deviation, decreasing allergic response in C57BL/6 mice
	
	OVA	Anti-allergic (78)	Pam3CSK4 (TLR2 agonist) reduced IL-4 and IL-5 secretion, whereas promoted Th1-associated cytokines

TLR3	OVA	Pro-allergic (97)	TLR3 agonist poly(I:C) with inhaled allergen leads to the development of allergic airway
	
	OVA	Anti-allergic (100)	Simultaneous engagement of TLR3 and TLR7 by viral components prevented airway hyperresponsiveness and suppressed established asthma

TLR5	OVA	Pro-allergic (90)	Nasal administration of OVA along with flagellin induced strong airway allergic responses
	
	OVA or HDM	Anti-allergic (93)	*Vibrio vulnificus*-derived flagellin B (FlaB) increases regulatory DCs Tregs, suppressing Th2 response

TLR7/8	HDM antigen followed by rhinovirus	Anti-allergic (106)	Increased levels of the Th2-priming cytokines IL-25 and TSLP in allergic TLR7^−/−^ BALB/c mice infected with RV
	
	OVA	Anti-allergic (107)	Resiquimod strongly suppressed Th2 cytokines
	
	Birch pollen extract	Anti-allergic (108)	Both prophylactic and therapeutic effects on allergic asthma
	
	Der p 2 or OVA	Anti-allergic (110)	A novel TLR7 ligand, arrested Th2-mediated airway inflammation *via* IL-10 and IFNγ
	
	OVA	Anti-allergic (109)	A novel TLR7 downregulated Th17 and Th2 responses
	
	OVA	Anti-allergic (111)	R848 induced Treg-mediated suppression of established asthma *via* TGF-β
	
	OVA	Anti-allergic (112)	TLR7 agonist (AZ12441970) *via* stimulation of type I interferon (IFN) inhibits Th2 immune responses

TLR9	Triple allergens (OVA, cockroach, HDM)	Anti-allergic (113)	CpG-ODN, TLR9 agonists, decreased allergen-specific IgE, eosinophils, and Th2-associated cytokines
	
	OVA	Anti-allergic (91)	CpG induced a low number of eosinophils in the BAL, predominance of CD8 T cells
	
	OVA	Anti-allergic (8, 119)	CpG ODNs may inhibit established Th2 immune responses through IFN-γ and IL-10 production

To investigate the relationship between TLRs and asthma, studies were undertaken to compare the expression of TLRs in PBMCs of asthmatic patients versus healthy subjects. It was found that the TLR1 and TLR2 expression in asthmatic patients is significantly higher while TLR6 expression is lower when compared to healthy subjects (84). Interestingly, TLR6 polymorphism has been associated with protection against clinical asthma (85). Studies in mice indicated that TLR2/6 agonists attenuate chronic allergic airway inflammation (86, 87). Another study assessed the effect of a synthetic TLR2/6 agonist, bisacyloxypropylcysteine polyethylene glycol conjugate on chronic allergic airway inflammation induced by allergen extract from Timothy grass pollen. The level of eosinophils, IL-5, IL-4, eotaxin-2, and RANTES in BALF was decreased after administration of the synthetic TLR2/6 agonist while the level of CD4^+^ Foxp3^+^ Treg cells and Th1 cells were unaffected suggesting that TLR2/6 agonist inhibits Th2-dominated immune response by another mechanism (86). Interestingly, Moreira et al. showed that stimulation of TLR6 on macrophages and DCs induced IL-23 production and directed naive CD4^+^ T cells toward Th17 population. Indeed, lung levels of IL-23 and IL-17 in *Tlr6^–/–^* C57BL/6 mice were markedly lower than wild-type allergic mice. Accordingly, administration of exogenous IL-23 restored the production of IL-17 and attenuated allergic responses as compared to the untreated allergic TLR6^–/–^ mice (88).

The pro- or anti-allergic effects of TLRs agonists might also rely on the type of allergen, dose, adjuvant used for sensitization and concomitant activation of TLRs by different agonists. For instance, divergent results were obtained with commercial LPS when OVA or *Blomia tropicalis* mite extract were used as allergens (89). It was found that commercial LPS, dampened allergic sensitization in the OVA-model while it induced Th17-type airway neutrophilic inflammation in *Blomia tropicalis* model (89). The reason for this became apparent when it was found that commercial LPS endotoxin is contaminated with lipid-associated proteins that activate TLR2. Thus, in *Blomia tropicalis* model concomitant activation of TLR2 and TLR4 dampen Th2 sensitization and boost Th17 sensitization while in the OVA model concomitant activation of TLR4 and TLR2 only hampers Th2 sensitization without shifting toward Th17 sensitization (89). Regarding the effects of different TLR2 agonists, it has been shown that tripalmitoyl-*S*-glycero-Cys-(Lys)4 (Pam3CSK4) which activates TLR2/1 potentiates FcεRI induced release of cytokines such as IL-8 from human mast cells (MCs), whereas PGN a TLR2/6 agonist is unable to stimulate this pathway (77) suggesting that differential dimerization of TLR2 with TLR1 or TLR6 can also play an important role.

## TLR5 and Asthma

A common microbial product found in house dust that could play a role in asthma is the bacterial protein flagellin, which activates TLR5 (90). It has been reported that asthmatic patients have higher serum levels of flagellin-specific antibodies when compared to non-asthmatic individuals (68, 90). Wilson et al. showed that TLR5 is required for strong priming of allergic responses induced by house dust extracts. Importantly, nasal administration of OVA accompanied by flagellin, contrary to OVA alone that induces tolerance, resulted in strong airway allergic responses (90). Also, experimental asthma could be established when OVA allergen is administrated together with flagellin as an adjuvant even in *Tlr4^−/−^* C57BL/6 and BALB/c mice (90). These results indicate that flagellin signaling *via* TLR5 in airway cells acts as a Th2 adjuvant. As discussed for TLR4, it was found that the pro-Th2 activity of flagellin involves the contribution of hematopoietic and non-HPCs, including lung ECs that produce cytokines, such as IL-1α, IL-1β, IL-6, TSLP, TNFα, and release of IL-33 (90, 91). Besides that, MyD88-dependent and independent signals, likely from IL-1R, IL-33R, and TSLP, respectively, were found to be required in cDCs for promotion of the early IL-4 response by CD4 T cells in response to flagellin (91). Strikingly, patients with severe asthma (Th17-type) express lower level of TLR5 on ECs of bronchi while TLR5 expression in patients with moderate asthma is similar to healthy controls (92). However, in opposition to the pro-Th2 allergic effect of TLR5, it was recently reported that administration of flagellin inhibited experimental asthma in therapeutic doses (93). These conflicting results appear to be related to the dose of flagellin used, low dose being pro-allergic and high dose anti-allergic. In addition, Shim et al. indicated that *Vibrio vulnificus*-derived flagellin B (FlaB) by acting on regulatory DCs and Treg cells suppressed Th2 response induced by OVA or HDM (93). Moreover, it was demonstrated that bone marrow-derived DCs stimulated by a fusion protein composed of OVA-flagellin resulted in the production of IL-10 (94, 95) that, in turn, inhibited type-2 cytokine production *in vitro* (95). Also, adoptive transfer of DCs exposed to OVA/FlaB prevented OVA-induced asthma. The role of CD25^+^ Treg cells in the inhibition of asthma was strengthened since depletion of CD25^+^ cells abrogated the suppressive activity of FlaB (93). Interestingly, the levels of TGF-β and IL-10 production were increased in DCs of asthmatic patients exposed to FlaB and TLR5 transcript in asthmatic DCs was increased after exposure to FlaB, reinforcing that TLR5 agonist could upregulate anti-inflammatory cytokines in asthmatic patients (93).

Collectively, the role of TLRs expressed on plasma membrane remains a controversial issue in asthma that requires more investigation.

## Endosomal TLRs (TLR3, 7, 8, and 9) and Asthma

Besides TLRs expressed on cell membrane, a group of endosomal TLRs agonists have gained increased consideration (Table 1). The TLR3, TLR7, TLR8, and TLR9 recognize nucleic acids and their stimulation result in the production of type I IFNs. TLR3 recognizes double-stranded RNA (dsRNA), TLR7, and TLR8 recognize single stranded RNA and TLR9 recognizes DNA (96).

## TLR3 and Asthma

Viral infections are known to exacerbate pulmonary allergic responses through amplification of Th2 cytokines and eosinophil infiltration (97). The possible mechanism by which viruses increase airway inflammation is activation of TLR3 *via* viral dsRNA (97). Experimentally, it was found that administration of synthetic TLR3 agonist poly(I:C) with inhaled allergen leads to the development of allergic airway (98). In contrast, mice treated with allergen along with TLR7/8 ligand (R848) prevented asthma development (98). Interestingly, simultaneous engagement of TLR3 and TLR7 by viral components prevented airway hyperresponsiveness and suppressed established asthma (99).

TSLP released by airway ECs primes DCs to Th2 cell differentiation (100). However, human DCs stimulated with TLR3 agonist plus TSLP favor Th17 cells differentiation, suggesting that viral infections acting through TLR3 stimulation of DCs might favor Th17 cell development and neutrophilic inflammation of asthmatic patients (100). Another mechanism by which viruses could induce neutrophilic inflammation is by stimulation of smooth muscle cells (101, 102). The stimulation of alveolar and bronchial smooth muscle cells with TLR2 (PGN), TLR3 (dsRNA), and TLR4 (LPS) ligands resulted in the release of CXCL8, a neutrophil-attracting chemokine. Among these agonists, dsRNA was the most potent inducer of CXCL8 (101, 102). Bronchial ECs might also contribute to neutrophilic inflammation since it was found that poly(I:C), a synthetic analog of viral dsRNA, enhanced the production of IL-6, IL-8, TNFα, and RANTES by human ECs in a dose-dependent manner (103). Therefore, all these airway cell types might exacerbate airway inflammation of asthmatics during viral infections by activating endosomal TLRs.

## TLR7 and Asthma

TLR7 has been also investigated and clinical studies support the protective effects of TLR7 in asthma since the TLR7 expression in bronchial epithelial biopsy of patients with severe asthma is markedly lower than healthy persons (92). Keeping with this, it was found that PBMCs of adolescents who suffer from asthma have low level of TLR7 expression and function and this might be a possible explanation for susceptibility to respiratory viral infections, which are a major cause of asthma exacerbations in children and adults (104). Recently, the effect of imiquimod (R837), a TLR7 agonist, on human airway and OVA-induced airway inflammation in guinea pigs was examined (105). The imiquimod (R837) through a nitric oxide-dependent mechanism not only relaxed the contraction of methacholine-induced human airways *in vitro* but also suppressed guinea pig airway inflammation *in vivo*. Application of TLR7 antagonist and nitric oxide inhibitor abolished this effect, indicating that nitric oxide plays a critical role in airway relaxation (105). Interestingly, the TLR8 agonists (polyuridylic acid and polyadenylic acid) also relaxed human airways, but the effect was nitric oxide independent (105). In fact, experiments performed *in vitro* suggest that stimulation of TLR7 in airway nerves results in bronchodilatation of both human and animal airways through nitric oxide production (105). The role of TLR7 in murine experimental asthma and viral-induced asthma exacerbation has been also investigated (106). Hatchwell et al. found that lack of TLR7 in HDM-induced allergic mice results in acceleration of rhinovirus (RV1B) replication, which in turn supports eosinophilic inflammation and airways hyper reactivity (106). Interestingly, they revealed that TLR7 expression in the lung of mice exposed to HDM or treated with IL-5 is suppressed, paving the way for respiratory viral infection (106).

An *in vivo* study using the OVA-model compared the anti-allergic effect of different TLR agonists administered by intranasal route (107). It was found that among all TLRs agonist tested, stimulation of TLR7 by Resiquimod was the most effective (107). Importantly, the suppressive effect on asthma by TLR7 agonists was also obtained when administered by intraperitoneal or epicutaneous routes (99, 108, 109). For instance, epicutaneous administration of Resiquimod (R848) together with birch pollen extract had both prophylactic and therapeutic effects on allergic asthma in a murine model (108). The therapeutic effects of TLR7 agonist have been evaluated by Nencini et al. that reported that conjugation of OH-modified adenine, a novel TLR7 ligand, with both Der p 2 (Der p 2-Conj) and OVA (OVA-Conj) diminished Th2-mediated airway inflammation in an IL-10 and IFN-γ-dependent manner (110), although the exact mechanisms by which TLR7 agonists forestall asthma development are still elusive. It appears that each synthetic agonist exploits distinct mechanism and in this context, activation of TLR7 by 9-benzyl-2-butoxy-8-hydroxy adenine, a ligand for TLR7, could attenuate murine asthma restraining Th17 and Th2 responses (109), whereas R848 arrests the symptoms of established asthma through Treg cells (111). Recently, a novel TLR7 agonist has been shown to inhibit murine allergic airways responses *via* type I IFN (112).

## TLR9 and Asthma

In a protocol of severe form asthma induced in mice by using triple allergens, such as OVA, cockroach extracts, and HDM, the effects of endosomal TLRs agonists were compared with dexamethasone (113). In this model, dexamethasone treatment was ineffective while resiquimod and CpG-ODN, TLR7 and TLR9 agonists, respectively, were effective in decreasing allergen-specific IgE, eosinophils, and Th2-associated cytokines (113). A double-blind, randomized trial study was conducted to investigate the efficacy of a novel TLR9 agonist known as QbG10 (bacteriophage Qbeta-derived virus-like particle with CpG-motif G10 inside). It was shown that TLR9 stimulation efficiently controls asthma manifestations (114).

In conclusion, as discussed for plasma membrane TLRs, endosomal TLRs agonists might prevent, suppress, or exacerbate asthma depending on the time, cell type engaged, and route of administration. It is likely that different anti-allergic mechanisms are triggered by TLR3, TLR7, TLR8, TLR9 agonists that might include the production of class I IFNs that counteract Th2-biased immune responses (115, 116) or immune-deviation toward Th1 response in the lung (117, 118). CpG-ODN stimulates DCs and alveolar macrophages to produce IL-12 that is essential for the innate phase of IFN-γ production and consequently Th1 polarization. Mice sensitized with OVA plus CpG showed a low number of eosinophils in the BAL, predominance of CD8 T cells, monocytes, and NK cells and high levels of OVA-specific IgG2c in serum when compared to control group (91). However, other studies indicated that type I or type II IFNs are dispensable for the inhibitory effect of CpG-ODN on asthma (119). Mirotti et al. and others indicated that IL-10 induced by CpG-ODN is a key cytokine for the suppression of allergic inflammation (8, 119).

## TLRs Signaling in Early and Late-Phase Asthmatic Reactions

Herxheimer was the first to describe two distinct components in the obstructive response to inhaled allergens, which he named the immediate and late reaction (120). The early-phase bronchoconstrictor response involves lung resident cells such as the MCs and anaphylactic antibodies leading to MC degranulation and the release of inflammatory mediators, such as histamine, prostaglandins, and leukotrienes, as well as cytokines, chemokines, and enzymes that are responsible for the allergic/anaphylactic symptoms (121, 122) while the late-phase reaction refers to bronchoconstriction taking place approximately 3–8 h after allergen inhalation that is associated with recruitment of T cells, eosinophils, neutrophils, and basophils (123).

Toll-like receptors signaling could affect these responses by interfering with immunoglobulin isotype switching in B cells or by interfering in cells that participate in allergic inflammation.

Regarding B cells, CpG-oligonucleotides and LPS have been shown to modulate B cell class switching (124). It was demonstrated that CpG induce class switching of murine B cells to IgG isotypes *via* TLR9 signaling and MyD88 pathway (125). Moreover, it was shown that CpG could inhibit class-switching toward IgE and to IgG1 (126). However, besides IgE, anaphylactic reactions could be also mediated by IgG antibodies that bind to IgG receptors on MCs and other cell types such as basophils, neutrophils, and macrophages (127).

Mast cells express most TLRs and actively participate in allergic responses through TLRs-induced cytokine and chemokine secretion (128). Stimulation of TLR3 on human MCs resulted in type I IFN production (128), while stimulation of TLR4 and 6 increased IL-13 release (129). However, although TLRs signaling in human MCs increases cytokine release it does not induce degranulation even in the presence of IgE (129). Stimulation of TLR4 on MC exacerbates murine experimental asthma (130) and upon LPS inhalation IL-5 production increases (131). The role of TLR4 on murine MCs was highlighted by experiments with adoptive transfer of bone marrow-derived MCs from wild-type mice to OVA-sensitized MCs-deficient mice. With cell transfers of TLR4-deficient MCs, mice developed Th2 responses and eosinophilia while with TLR4-deficient MCs they failed to develop it (131).

Eosinophils are key cells in allergic responses (132, 133). Several studies have shown that stimulation of TLRs, especially endosomal TLRs on eosinophils, results in increase of their activity and cytokine release (132, 133). For example, human eosinophils increase their adhesion molecule CD11b and IL-8 secretion upon exposure to R-837 and CpG DNA, suggesting TLR7/8 and TLR9 in human eosinophil could be responsible for asthma exacerbation by viral infections (134).

Basophils and neutrophils also contribute in atopic and non-atopic asthma, respectively. Recently, Suurmond et al. reported that activation of basophil TLRs supports allergic responses by increasing the production of IL-4 and IL-13 along with IL-8 and RANTES (135). The involvement of neutrophils in severe asthma has been studied and recently, it was reported that HDM *via* stimulation of TLR4 on neutrophils impairs neutrophil apoptosis (136). Furthermore, neutrophil TLR4 activation during infection with respiratory syncytial virus potentiates airway inflammation *via* production of heat shock protein 72 (137).

All these reports indicate that TLRs signaling might exacerbate asthma. However, in Th2 cells, LPS reduced IL-4 production (138). Keeping with this, we have previously shown that systemic LPS administration blocks airway allergic inflammation and passive cutaneous anaphylaxis (PCA) *via* nitric oxide synthase 2 activity (6). Since in the PCA assay, anaphylactic antibodies are passively transferred, the inhibition of anaphylaxis could be attributed to blockage of MCs or other cells in releasing inflammatory mediators. Studies performed *in vitro* and *in vivo* revealed that NO inhibit MC degranulation and MC-dependent cellular inflammation (139). In the same vein, we postulate that LPS could inhibit exocytosis of other cells involved in anaphylaxis such as basophils. In addition, the induction of type 1 or type 2 IFNs by TLRs agonists signaling in monocyte, macrophages, DCs and NK cells might counterbalance the pro-allergic effect of TLRs signaling in other airway cell types, such as ECs, MCs, eosinophils, basophils, and neutrophils, during the late-phase reaction.

## Concluding Remarks

The data provided from human and animal studies indicate that the influence of TLRs agonists on asthma outcome might depend on the cell type that is activated (hematopoietic versus non-HPCs), nature of the allergen (HDM versus OVA), and the route of administration (airway versus subcutaneous/peritoneal). Here, we discussed that TLRs could suppress, exacerbate, or contribute to asthma pathogenesis. Therefore, the use of TLRs agonists for treatment of asthma must be carefully evaluated and precisely designed to achieve therapeutic value.

## Author Contributions

Both authors contributed equally.

## Conflict of Interest Statement

The authors declare that the research was conducted in the absence of any commercial or financial relationships that could be construed as a potential conflict of interest.
